# Polysialic acid and Siglec-E orchestrate negative feedback regulation of microglia activation

**DOI:** 10.1007/s00018-020-03601-z

**Published:** 2020-07-28

**Authors:** Hauke Thiesler, Julia Beimdiek, Herbert Hildebrandt

**Affiliations:** 1 Institute of Clinical Biochemistry, Hannover Medical School, Carl-Neuberg-Straße 1, 30625 Hanover, Germany; 2 Center for Systems Neuroscience Hannover (ZSN), Bünteweg 2, 30559 Hanover, Germany

**Keywords:** Immune balance, Inflammatory activation, Innate immune response, Traumatic brain injury, Sialic acid-binding immunoglobulin-like lectins

## Abstract

Polysialic acid (polySia) emerges as a novel regulator of microglia activity. We recently identified polysialylated proteins in the Golgi compartment of murine microglia that are released in response to inflammatory stimulation. Since exogenously added polySia is able to attenuate the inflammatory response, we proposed that the release of polysialylated proteins constitutes a mechanism for negative feedback regulation of microglia activation. Here, we demonstrate that translocation of polySia from the Golgi to the cell surface can be induced by calcium depletion of the Golgi compartment and that polysialylated proteins are continuously released for at least 24 h after the onset of inflammatory stimulation. The latter was unexpected, because polySia signals detected by immunocytochemistry are rapidly depleted. However, it indicates that the amount of released polySia is much higher than anticipated based on immunostaining. This may be crucial for microglial responses during traumatic brain injury (TBI), as we detected polySia signals in activated microglia around a stab wound in the adult mouse brain. In BV2 microglia, the putative polySia receptor Siglec-E is internalized during lipopolysaccharide (LPS)-induced activation and in response to polySia exposure, indicating interaction. Correspondingly, CRISPR/Cas9-mediated Siglec-E knockout prevents inhibition of pro inflammatory activation by exogenously added polySia and leads to a strong increase of the LPS response. A comparable increase of LPS-induced activation has been observed in microglia with abolished polySia synthesis. Together, these results indicate that the release of the microglia-intrinsic polySia pool, as implicated in TBI, inhibits the inflammatory response by acting as a *trans*-activating ligand of Siglec-E.

**Electronic supplementary material:**

The online version of this article (10.1007/s00018-020-03601-z) contains supplementary material, which is available to authorized users.

## Introduction

The glycan polysialic acid (polySia) is the α2,8-linked homopolymer of N-acetylneuraminic acid (Neu5Ac), the most common sialic acid in vertebrates [1]. PolySia is mainly known as a posttranslational modification of the neural cell adhesion molecule (NCAM). PolySia on NCAM is presented at the cell surface and plays a major role during brain development by modulating NCAM binding and attenuating other cell surface interactions [2, 3]. Recently, however, we identified a pool of polySia in primary and stem cell-derived murine microglia and human THP-1 macrophages occurring on two other, unrelated protein carriers, namely neuropilin 2 (NRP2) and the E-selectin ligand 1 (ESL-1, a.k.a. GLG1) [4, 5]. Surprisingly, this polySia pool is not found at the cell surface, but restricted to the Golgi compartment and in contrast to polySia on NCAM, which can be synthesized by the two Golgi-resident polysialyltransferases ST8SIA2 and ST8SIA4 [6], it is exclusively produced by ST8SIA4 [4]. In acute brain slice cultures, Golgi-localized polySia appears during injury-induced activation of microglia, and inflammatory activation by stimulation with bacterial lipopolysaccharide (LPS) causes a rapid loss of cell-associated polySia, because polySia-NRP2 and polySia-ESL-1 are no longer retained in the Golgi, but translocated to the cell surface and released by ectodomain shedding [5].

Application of soluble, free or protein-bound polySia attenuates proinflammatory activation of primary and stem cell-derived murine microglia, murine BV2 microglia, and human THP-1 macrophages [4, 5, 7–9]. Based on this effect of exogenously applied polySia, we proposed that the release of the cell-intrinsic pool of polysialylated proteins provides negative feedback regulation of microglia and macrophage activation. This is supported by the observation that LPS-induced activation is potentiated in ST8SIA4-deficient primary murine microglia [4], because the deprivation of polySia synthesis and thereby the absence of polySia shedding may lead to a loss of feedback inhibition. However, the receptor responsible for polySia sensing in this assumed feedback loop remained elusive.

Sialylated glycans are recognized by members of the I-type lectin family called sialic acid-binding immunoglobulin-like lectins (Siglecs) that are primarily found on hematopoietic and immune cells [10–12]. Conventionally, Siglecs are divided into those that are structurally conserved across mammals, and the group of CD33 (Siglec-3)-related Siglecs that vary considerably between species. Siglecs are type I transmembrane proteins with an amino-terminal sialic acid-binding V-set domain. In their cytoplasmic domain, most of the CD33-related Siglecs have immunoreceptor tyrosine-based inhibitory motifs (ITIMs), which typically counteract activating signaling from other immune receptors [11, 13]. In the human system, the CD33-related macrophage- and microglia-specific Siglec-11 has been shown to bind polySia and to attenuate proinflammatory activation [8, 9, 14]. Siglec-11 has no murine orthologue [15], but glycan array binding revealed that murine Siglec-E, among a wide range of other sialoglycans, binds α2,8-linked di- and trisialic acid [16] and recent in vitro data indicated that polySia encapsulated *E. coli* K1 bind to Siglec-E as efficiently as to human Siglec-11 [17]. Moreover, lentiviral knockdown of Siglec-E abolished the responsiveness of LPS-induced microglia to a fraction of polySia with an average degree of polymerization of 20 sialic acid residues (avDP20) [9], but the authors emphasize that compared to Siglec-11-positive human THP-1 macrophages, about tenfold more avDP20 was needed to elicit the same inhibitory response in mouse microglia with uncompromised expression of Siglec-E. This conspicuous discrepancy raises doubts, if Siglec-E qualifies as a receptor of microglial polySia release.

In the current study, we address open questions concerning the mechanism of the LPS-induced discharge of polysialylated proteins from the Golgi of microglia and the time course of their release. In addition, we demonstrate a transient accumulation of polySia in injury-induced microglia in vivo and provide evidence that Siglec-E is involved in negative feedback inhibition by acting as a receptor for polySia on proteins shed by LPS-induced microglia.

## Materials and methods

### Cells, reagents and antibodies

BV2 microglia were kindly provided by Gerd Bicker, University of Veterinary Medicine, Hannover, Germany. If not indicated otherwise, BV2 cells were cultured in DMEM with 4.5-g/l glucose containing 100-µg/ml penicillin/streptomycin and supplemented with 2.5% fetal bovine serum (all from Gibco, Rockville, MD, USA). Passaging was performed at about 80% confluency and cells were detached mechanically with a cell scraper. All experiments performed in this study were obtained between passages 6 and 20. The plasmid pX330A-1 × 2 for CRISPR/spCas9-D10A-mediated knockout [18] was a kind gift from Takashi Yamamoto (Addgene, Watertown, MA, USA, plasmid #58766; https://n2t.net/addgene:58766; RRID:Addgene_58766). The plasmid DHC#4731 containing a P2A element followed by eGFP [19] was kindly provided by Dirk Heckl, Hannover Medical School, Hannover, Germany. Endosialidase was produced as described before [20]. Trisialic acid, i.e., α2,8-linked sialic acid with a degree of polymerization (DP) of 3, and tetrasialic acid (DP4) were from Nacalai Tesque (Kyoto, Japan); α2,8-linked polySia (colominic acid from *E. coli*) was from Sigma-Aldrich [St Louis, MO, USA, catalog no. (cat. #) C5762, lot no. 110M1383]. Streptavidin and biotin were from Vector Laboratories (Burlingame, CA, USA), Cy3-conjugated streptavidin from Rockland (Limerick, PA, USA). Lipopolysaccharide (LPS) extracted from *E. coli* serotype O127:B8, 4-chloro-*m*-cresol (4-C*m*C), TAK-242, 1,1′‐diheptyl‐4,4′‐bipyridinium dibromide (DHBP), genistein, and biotinyl tyramide were from Merck, Darmstadt, Germany.

The following monoclonal (mAb) or polyclonal (pAb) antibodies were used: Siglec-E-specific sheep pAb (kindly provided by Paul Crocker, University of Dundee, Scotland, 3.2 µg/ml), ESL-1-specific rabbit pAb (kindly provided by Martin Wild, Münster, Germany, 1:3000), polySia-specific mouse mAb 735 ([21]; produced in-house as described by Werneburg et al. [4] and used at 2 µg/ml for immunofluorescence or 1 µg/ml for immunoblotting), CD11b‐specific rat mAb (AbD Serotec, Raleigh, NC, USA, cat. #MCA74GA, 1:250), Iba1‐specific rabbit pAb (Wako Chemicals, Neuss, Germany, cat. #019‐19741, 1:300), giantin‐specific rabbit pAb (Covance, Denver, PA, USA, cat. #PRB‐114C, 1:10,000), NRP2‐specific rabbit mAb D39A5 (Cell Signaling, Beverly, MA, USA, cat. #3366S, 1:1000), EEA1 (early endosomal antigen 1)-specific antibody rabbit pAb (Santa Cruz Biotechnology, Santa Cruz, CA, cat. #sc-33585, 1:100).

Secondary antibodies were HRP-conjugated donkey anti-sheep IgG (Sigma-Aldrich, cat. #A-3415, 1:1000), Cy3-conjugated donkey anti-rat IgG (Merck, cat. #AP189C, 1:500), Alexa Fluor 647-conjugated donkey anti-rabbit IgG (Thermo Fisher Scientific, Waltham, MA, USA, cat. #A-31573, 1:500), Alexa Fluor 488-conjugated donkey anti-mouse IgG (Thermo Fisher Scientific, cat. #A21020, 1:500), Alexa Fluor 488-cojugated goat anti-mouse IgG (Thermo Fisher Scientific, cat. #A11029, 1:500), HRP-conjugated goat anti-rabbit IgG (Sigma-Aldrich cat. #6157, 1:15,000), and HRP-conjugated goat anti-mouse IgG (Southern Biotech, Birmingham, AL, USA, cat. #1010–05, 1:20,000).

### Immunocytochemistry

For immunocytochemistry, cells were seeded on glass coverslips in 24-well plates at a density of 20,000 cells per well. After a given treatment, as specified for each experiment separately, cells were washed once in PBS, fixed with 4% paraformaldehyde in PBS for 20 min at room temperature (RT) and stored in PBS at 4 °C. Immunofluorescence staining (IF) and embedding was performed as described by Werneburg et al. [4] with minor modifications by permeabilizing with 0.4% Triton X-100 in PBS for 15 min, blocking for 2 h at 37 °C, and washing three times with 0.1% Triton in PBS and once with water, before mounting.

For Siglec-E immunostaining in combination with other primary antibodies, biotin-tyramide signal amplification was used. Briefly, after permeabilization, peroxidase activity was blocked by incubation with 0.3% H_2_O_2_ in PBS for 15 min at RT. After washing, endogenous biotin was blocked by adding streptavidin and, after further washing, biotin (1:10 in PBS, 15 min at RT, each). After another washing step, cells were incubated with primary antibodies as described above, and for 45 min with HRP-conjugated anti-sheep IgG for the detection of Siglec-E-specific antibody, followed by washing with TBS pH 7.6 and incubation in TBS with 0.1-M imidazole, 0.001% H_2_O_2_, and 2.5-µg/ml biotinyl tyramide for 10 min at RT. After three washes with PBS, enzymatically produced biotin precipitate was detected by Cy3-conjugated streptavidin (1:1.000 in PBS with 0.1% Triton X-100 and 2% BSA, 45-min RT), before fluorescently labeled secondary antibodies were added and cells were embedded, as described above.

Specificity of double- and triple-immunostaining procedures was controlled by omitting one of the primary or secondary antibodies at a time. For polySia immunoreactivity, specificity was additionally controlled by the loss of immunoreactivity after degradation of polySia with 6-µg/ml endosialidase applied during blocking. This enzyme degrades polySia with high specificity [20].

### Immunoaffinity chromatography

Stationary phase consisting of polySia-specific mAb 735 coupled to protein A sepharose beads (GE Healthcare, Amersham, UK) was prepared using standard protocols [22]. A C16/20 column (GE Healthcare) was packed with 3.2-ml beads coupled with 2.1-mg antibody per ml beads (bed volume) and chromatography was performed at RT on an Äkta Pure 25 protein purification system (GE Healthcare) at a flow rate of 0.4 ml/min with 20-mM Tris–HCl, pH 8.0, 10-mM MgCl_2_ containing 90-mM NaCl for loading and 2-M NaCl for elution. Conductivity and absorption at 214 and 280 nm were recorded.

Supernatant of LPS-induced BV cells cultured in serum-free medium was passed through a 0.22-µm syringe filter prior to loading and following loading to the column elution was started after a stable baseline was obtained. For subsequent analysis by immunoblotting (see below), fractions of 0.4 ml were collected and 1.6-ml propanone per fraction was added to precipitate proteins overnight at -20 °C. After centrifugation at 16,000×*g* for 15 min, pellets were air dried and reconstituted in 20-µl Laemmli buffer.

### Immunoprecipitation and immunoblotting

Immunoprecipitation (IP) of polySia from cell lysates using M-280 tosyl-activated Dynabeads (Thermo Fisher Scientific) covalently coupled to mAb 735, SDS-PAGE and analyses of IP fractions by immunoblotting and enhanced chemiluminescence detection were performed as described by Werneburg et al. [5]. Fractions obtained by immunoaffinity chromatography were blotted on nitrocellulose membrane (GE Healthcare) and analyzed with the Odyssey Infrared Imaging System (LI-COR Biosciences, Bad Homburg, Germany) as described previously [23]. For enzymatic removal of polySia prior to SDS-PAGE, samples in Laemmli buffer were treated with 6-µg/ml endosialidase for 45 min at 37 °C.

### Anion exchange chromatography

Polysialic acid was separated on the Äkta Pure 25 system equipped with a 22 × 50 guard column followed by a DNAPac PA-100 column 22 × 250 (Thermo Fisher Scientific). Chromatography was performed at RT with a flow rate of 3.5 ml/min using 10-mM Tris–HCl pH 8.0 during loading. Elution was performed in 10-mM Tris–HCl pH 8.0 with consecutive steps of linear NaCl gradients and steady concentrations as follows: 4-ml 0–70 mM, 44-ml 70–180 mM, 0.5-ml 180 mM, 16-ml 180–220 mM, 48-ml 220–280%, 40-ml 280–320 mM, 80-ml 320–355 mM, and 40-ml 355–385 mM, 40-ml 385–410 mM, 44-ml 410–432 mM, 40-ml 432–447 mM, 40 ml 447–458 mM, 40-ml 458–466 mM, 40-ml 466–470 mM, 40-ml 470–472 mM and 45-ml 1 M NaCl. Conductivity and absorption at 214 and 280 nm were recorded.

### Brain sections

To conserve animals, sections of mouse brains with cortical lesions were drawn from a control group of animals used in a prior study, which was designed to examine drug effects on epileptogenesis induced by intrahippocampal kainate injection [24]. Sections were kindly provided by Wolfgang Löscher, University of Veterinary Medicine, Hanover, Germany. The animal experiments were performed according to the EU council directive 2010/63/EU and the German Law on Animal Protection. Ethical approval was granted by the Lower Saxony State Office for Consumer Protection and Food Safety (LAVES, project number 14/1659). As described in detail elsewhere [24], 8- to 10-week-old male NMRI mice were anesthetized with chloral hydrate (500 mg/kg i.p) and received a unilateral stereotactic injection of kainate (1 nM in 50-nl saline) through the cortex into the CA1 sector of the dorsal hippocampus. Injections were carried out with a 0.5-μl microsyringe over 60 s and after injection, the syringe was kept in place for another 2 min to limit reflux along the wound channel. In addition, mice received daily i.p. injections of saline. Seven days after the brain injection, and before the onset of clinical seizures [25], mice were perfused and paraffin-embedded brains were sectioned at 3 µm for use in immunohistochemistry.

### Immunohistochemistry

Paraffin sections were rehydrated and antigen retrieval was performed in 400-mM Tris–HCl pH 9.0 with 1-mM EDTA and 0.05% Tween-20 at 90 °C for 10 min. After permeabilization with 0.4% Triton X-100 in PBS for 30 min and blocking with 2% BSA, 0.1% Triton in PBS for 3 h at 37 °C, primary antibodies were incubated in blocking buffer at 4 °C overnight. All other steps and specificity controls were carried out as described for immunocytochemistry, except for the endosialidase treatment, which was performed with 10 µg/ml.

### Microscopy, image acquisition, cell counting and densitometry

Microscopy was performed with Axio Observer.Z1 equipped with an ApoTome module for structured illumination, AxioCam MRm digital camera, and Zen 2012 (blue edition) software (Carl Zeiss Microscopy). A 20 × Plan‐Apochromat objective with a numerical aperture of 0.8 was used to acquire optical sections of 1.62-μm (488 channels), 1.75-μm (568 channels) or 2.09-µm thickness (647 channels), and five optical sections were merged to a maximal intensity projection, respectively. For 3D reconstruction, stacks of 40 optical sections were obtained using a 63 × Plan-Apochromat oil immersion objective with a numerical aperture of 1.4. Identical settings were used for all samples within one experimental setting. Images were arranged using PowerPoint (Microsoft Office 2010). For the evaluation of immunostained cells in cultures, cell counts were performed on randomly chosen frames as specified in the respective figure legends. For the evaluation of Siglec-E immunoreactivity, cells were encircled manually and signal intensities were determined using the polygon contour measurement tool of the ZEN software. For the evaluation of Iba-1- and polySia-positive cells on brain sections, immuno-positive cells were counted in bins of 0–50, 50–200 and 200–400 µm away from the wound channel.

### Generation of Siglec-E knockout cells

The CRISPR/spCas9-D10A strategy [18, 26] was used to generate Siglec-E knockout cells. The all-in-one CRSIPR/spCas9-D10A vector pX330A-1 × 2 (Addgene plasmid # 58766) was modified by insertion of a P2A element followed by eGFP in C-terminal orientation. The latter was obtained from plasmid DHC#4731 as a template by overlap extension PCR with the primers 5′- GGAAGAGAATGCTGGCCTCT -3′ and 5′- AATCCAGAATTCGATTATCGATTTAACGC -3′ containing recognition sequences for BsmI and EcoRI. In addition, the following guide RNAs (gRNA) targeting the sialic acid-binding V-domain of Siglec-E located within the first exon were inserted: 5′- CACCGCAGACGCAAAGATTCCATCG -3′ (leading strand gRNA1), 5′- AAACCGATGGAATCTTTGCGTCTGC -3′ (reverse strand gRNA1), 5′- CACCGTGTACCAGAATCCATGAACT -3′ (leading strand gRNA2), 5′- AAACAGTTCATGGATTCTGGTACAC -3′ (reverse strand gRNA2).

To transfect BV2 cells, electroporation with the Neon Nucleofector (Thermo Fisher Scientific) was used according to the manufacturer’s instructions. 1 × 10^5^ cells were treated with a 30-ms pulse of 1325 V followed by incubation for 12 h in conditioned medium consisting of supernatant from 1 × 10^5^ BV2 cells per ml, cultured for 24 h in the absence of penicillin and streptomycin, which was filtered through a 0.22-µm syringe filter and supplemented with FCS to a final concentration of 7.5%. Subsequently, cells were cultured in conditioned medium with penicillin and streptomycin supplemented with 5% FCS for 3 days and 2.5% FCS for 7 days, followed by standard cultivation conditions. GFP-positive cells were identified, clones were isolated by limited dilution and Siglec-E mutations were assessed by sequencing of genomic PCR products obtained with primers flanking the targeted site (forward: 5′- CAGTTTTAGCTGGACATGCTG -3′, reverse: 5′- CGGGTTTCCTTCACTGCTT -3′).

### Nitrite Assay

Nitric oxide (NO) production by LPS treatment was evaluated by the colorimetric Griess assay, detecting the stable NO breakdown product nitrite in cell culture supernatants. The assay was performed as described before [7], with the following modifications: 5 × 10^4^ cells were seeded in 96-well plates. After adherence for 4 h, the medium was changed and cells were cultured for 2 h in the presence of 60-µM minocycline, where indicated. After another medium change, cells were kept for 24 h in 200-µl medium for further treatment (see results), before 150 µl of cell culture supernatant were collected and centrifuged at 3000×*g* for 5 min. Nitrite concentrations were assessed by reacting 70 µl of the supernatant for 20 min with 70-µl Griess reagent consisting of 1% (4-[(4-aminobenzene)sulfonyl]aniline), 0.05% *N*-1-napthylethylenediamine dihydrochloride and 2.5% HCl.

### RNA isolation and quantitative real-time RT-PCR

Total RNA was isolated using TRIzol (Thermo Fisher Scientific), and cDNA was generated as described [4] using RevertAid H Minus reverse transcriptase (Thermo Fisher Scientific). Quantitative real-time PCR of mouse Siglec-E, TNF and IL-6 was performed in 10-µl 1:2 diluted BIO SyGreen Lo-ROX mix (PCR Biosystems, London, UK) with ImageQuantQ3 and ImageQuant software (Thermo Fisher Scientific) using the comparative threshold cycle (Δ*C*_T_) method as described elsewhere [27] with HPRT and PPIA as reference genes [28]. The following primers were used: HPRT, 5′- TTCCTCATGGACTGATTATGGACA-3′ (forward) and 5′- AGAGGGCCACAATGTGATGG -3′ (reverse); PPIA, 5′- CCACAGTCGGAAATGGTGAT-3′ (forward) and 5′- TGCACTGCCAAGACTGAATG-3′ (reverse); TNF, 5′- CTGTAGCCCACGTCGTAGC-3′ (forward) and 5′- TTGAGATCCATGCCGTTG-3′ (reverse) [29]; IL-6, 5′-GGCCTTCCCTACTTCACAAG -3′ (forward) and 5′-ATTTCCACGATTTCCCAGAG -3′ (reverse) [30]; Siglec-E 5′-TGGTACAGGGAAGGAACCGA -3′ (forward) and 5′-GTGAGGGCTGTTACAACCAGA -3′ (reverse). The NCBI primer designing tool with Primer3 version 4.1 [31, 32] was used to select Siglec-E-specific primers.

### Statistics

Statistical analysis was performed using GraphPad Prism 7 software (GraphPad, San Diego, CA, USA). Unpaired Student’s *t*-test, one-way analysis of variance (ANOVA) followed by Tukey’s post hoc test, and mixed two-way ANOVA followed by Holm–Sidak post hoc test were applied as indicated. Normality and equality of variances were assessed by the Shapiro–Wilk and the Brown–Forsythe test, respectively.

## Results

### BV2 cells produce polySia on NRP2 and ESL-1

**Fig. 1:**
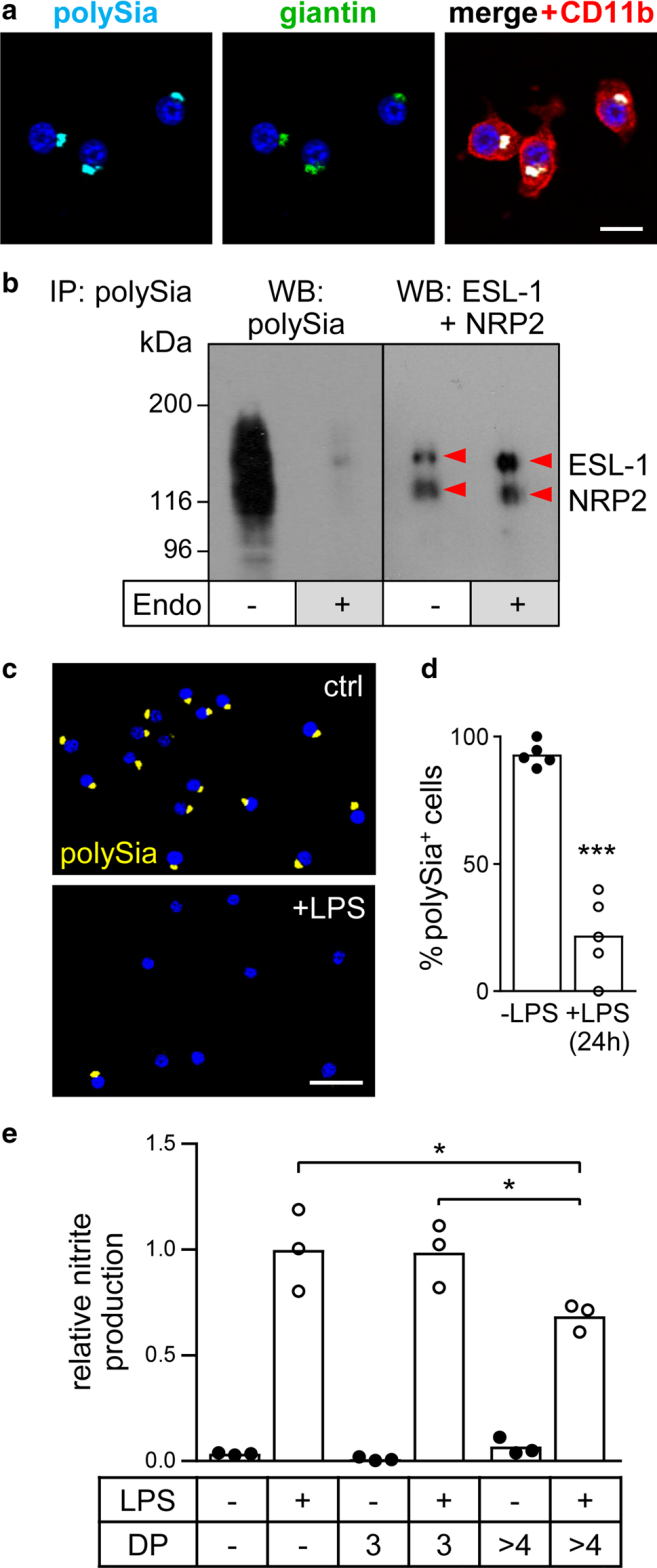
Validation of polySia expression and polysialylated proteins in BV2 microglia. **a** Immunofluorescence staining of polySia co-localized with the Golgi marker giantin. Cell shape is highlighted by co-staining with the microglia/macrophage marker CD11b. Nuclei were counterstained with DAPI (blue). Scale bar, 20 µm. **b** Immunoprecipitation (IP) of polysialylated proteins from lysate of 10^7^ BV2 cells using polySia-specific mAb 735-conjugated magnetic beads followed by Western blot (WB) detection with polySia-specific antibody (left), or by joint incubation with NRP2- and ESL-1-specific antibodies (right). Where indicated, IP fractions were treated with endosialidase (endo +), to remove polysialic acid. Protein bands were assigned according to the apparent molecular weights of NRP2 and ESL-1 as previously detected in primary and stem cell-derived murine microglia or in mouse brain tissue [5; see text for details]. **c** Compared to untreated controls (ctrl), incubation of BV2 cultures with 1-µg/ml LPS for 24 h leads to the loss of polySia signals in almost all cells. Nuclei were counterstained with DAPI (blue). Scale bar, 50 µm. **d** Loss of polySia-positive cells after LPS treatment, as indicated. Individual values and means of 5 evaluated frames per culture condition, with a minimum of 20 cells each are plotted and significant difference by two-tailed t-test is indicated (*** *p* < 0.001). **e** PolySia with DP > 4 but not trisialic acid (DP3) attenuates the LPS-induced production of NO. Nitrite levels in the supernatant of BV2 cells cultured for 24 h in the presence or absence of 30-nM trisialic acid (DP3), 500-ng/ml poly—Sia (approximately 30 nM, see text for details), and/or 1-µg/ml LPS, as indicated. Individual values and means from 3 independent treatments per group are plotted. One-way ANOVA indicated significant differences (*p* < 0.0001) and results from Tukey’s post hoc test are shown for comparisons between the LPS-treated groups (*p < 0.05)

The murine microglial cell line BV2 is a commonly used model system and suited to study inflammatory microglia activation [33]. As shown before [7], LPS-induced activation of these cells can be dose dependently inhibited by externally applied polySia. For the use of BV2 cells in the current study, we validated, if these cells also recapitulate the previously described features of primary and stem cell-derived microglia concerning the production of polySia [4, 5]. Immunofluorescence (IF) revealed that BV2 cells accumulate polySia in the Golgi compartment (Fig. 1a) and immunoprecipitation with polySia-specific antibody followed by Western blot analysis demonstrated the presence of the two polySia carrier proteins NRP2 and ESL-1 (Fig. 1b). When treated with the polySia degrading enzyme endosialidase, both proteins exhibited the small shift towards lower apparent molecular mass that is characteristic for the loss of polySia [5] (Fig. 1b). Moreover, BV2 cells show a depletion of the Golgi-resident polySia pool after proinflammatory stimulation with LPS for 24 h (Fig. 1c, d). Consistent with the previous results [7], the application of 500 ng/ml of polySia (colominic acid) caused a significant inhibition of LPS-induced NO production (Fig. 1e). As shown by HPLC analysis, the specific polySia batch used in the current study comprised chain lengths ranging from a degree of polymerization (DP) of 5 to DP > 100 with an average DP of about 50 (for a DP profile, see Online Resource 1). The effective molar concentration, therefore, was approximately 30 nM. In contrast to the effect of polySia, the same molar concentration of trisialic acid (DP3) was not able to dampen the inflammatory response (Fig. 1e). Although statistically not significant, the slight increase of NO production observed in BV2 cells treated with polySia alone might be caused by endotoxin contamination of the polySia preparation. Replication by an independent experiment, however, yielded no evidence for such an effect [nitrite levels relative to LPS-treated cells were 0.042 ± 0.011 for untreated and 0.046 ± 0.008 for polySia (> DP4) treated BV2 cells, respectively (means ± s.d., *n* = 4 independent cultures each, comparison by unpaired, two-sided *t* test, *p* = 0.56)]. Collectively, these results establish that BV2 cells qualify for studies on the mechanisms of polySia release and perception.

### Golgi retention and release of polySia are calcium sensitive

**Fig. 2:**
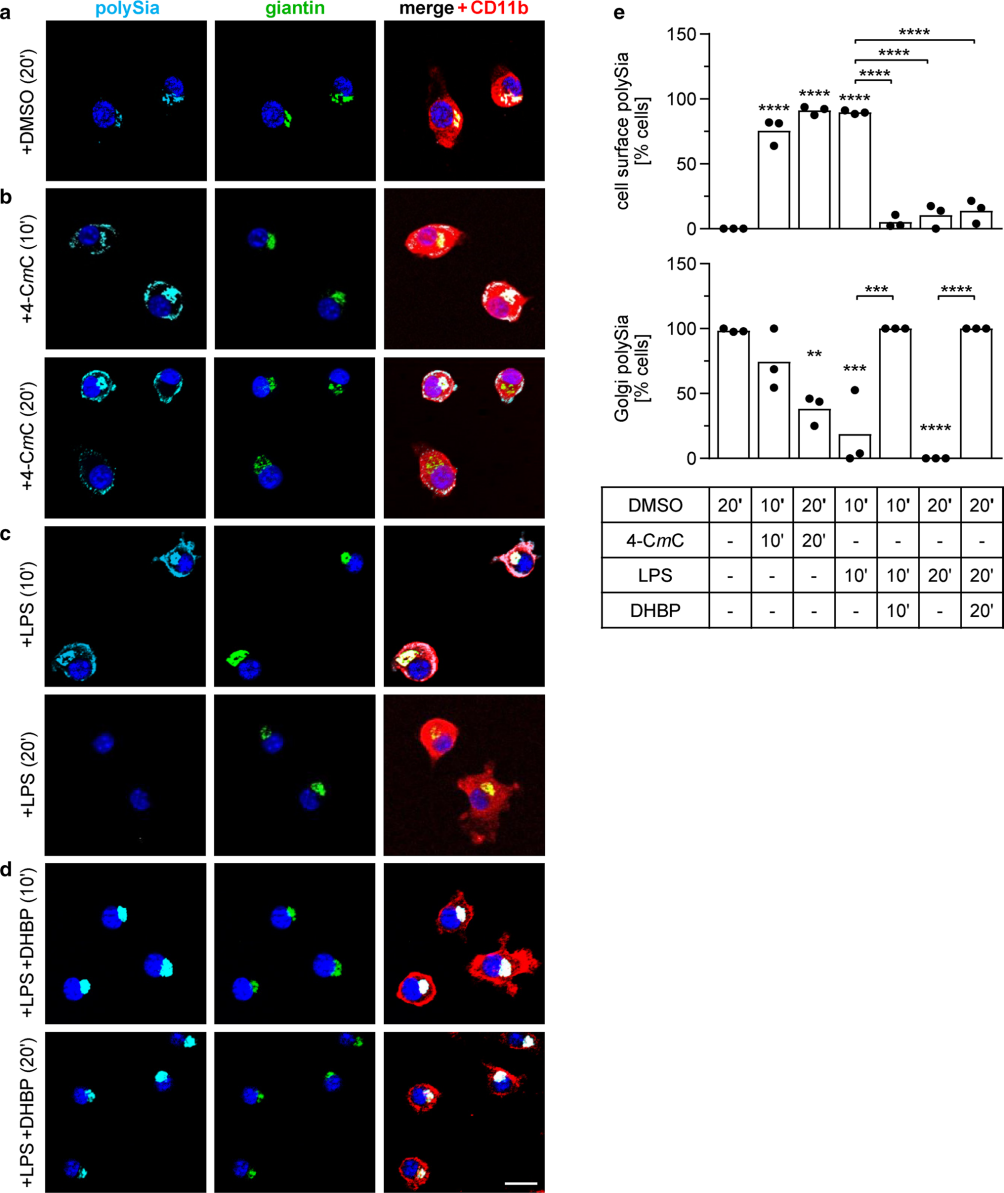
PolySia staining patterns in BV2 cells treated for 10 or 20 min with 1-µl/ml DMSO (**a**), 50-µM RyR agonist 4-C*m*C (**b**), 1-µg/ml LPS (**c**), or 1-µg/ml LPS together with 10-µM RyR antagonist DHBP (**d**), and quantitative assessment (**e**), as indicated. Per well, a minimum of 10 cells in 3 randomly selected frames with at least 3 cells each were evaluated and percentages of cells with detectable polySia signals at the cell surface (upper graph) or co-localized with the Golgi marker giantin (lower graph) were calculated. Individual values and means of 3 wells per condition are plotted. For each data set, one-way ANOVA indicated significant differences (*p* < 0.0001) and results from Tukey’s post hoc test are shown for comparisons against the DMSO control and for selected group comparisons (**p < 0.01, ***p < 0.001, ****p < 0.0001). 4-C*m*C and DHBP were added as 1-µl/ml stock solution in DMSO. Giantin and CD11b were co-stained to visualize the Golgi compartment and the cells’ shape, respectively. Nuclei were counterstained with DAPI (blue). Scale bar, 20 µm. See text for a description of treatment effects

A key feature of proinflammatory activation is the elevation of the cytosolic Ca^2+^ level by the depletion of the intracellular Ca^2+^ stores of the endoplasmic reticulum (ER) and Golgi compartment [34]. This can be mimicked by the pharmacological activation of ryanodine receptors (RyRs) [35]. Application of the RyR agonist 4-C*m*C for 10 min caused a translocation of polySia from the Golgi to the cell surface, which was comparable to the effect of LPS (Fig. 2b, c; for a quantitative assessment see Fig. 2e). After 20 min, however, the cell-associated polySia signals in LPS-treated cells had vanished completely; whereas, the 4-C*m*C-treated cells retained cell surface staining (Fig. 2b, c, e). Conversely, the RyR receptor antagonist DHBP prevented the LPS-induced translocation of the Golgi-resident polySia pool (Fig. 2d, e). This implies that the translocation of polysialylated proteins from the Golgi compartment to the cell surface in response to proinflammatory activation of microglia is triggered by lowering the calcium level within the Golgi compartment.

### Continuous release of polySia by LPS-induced BV2 cells

**Fig. 3:**
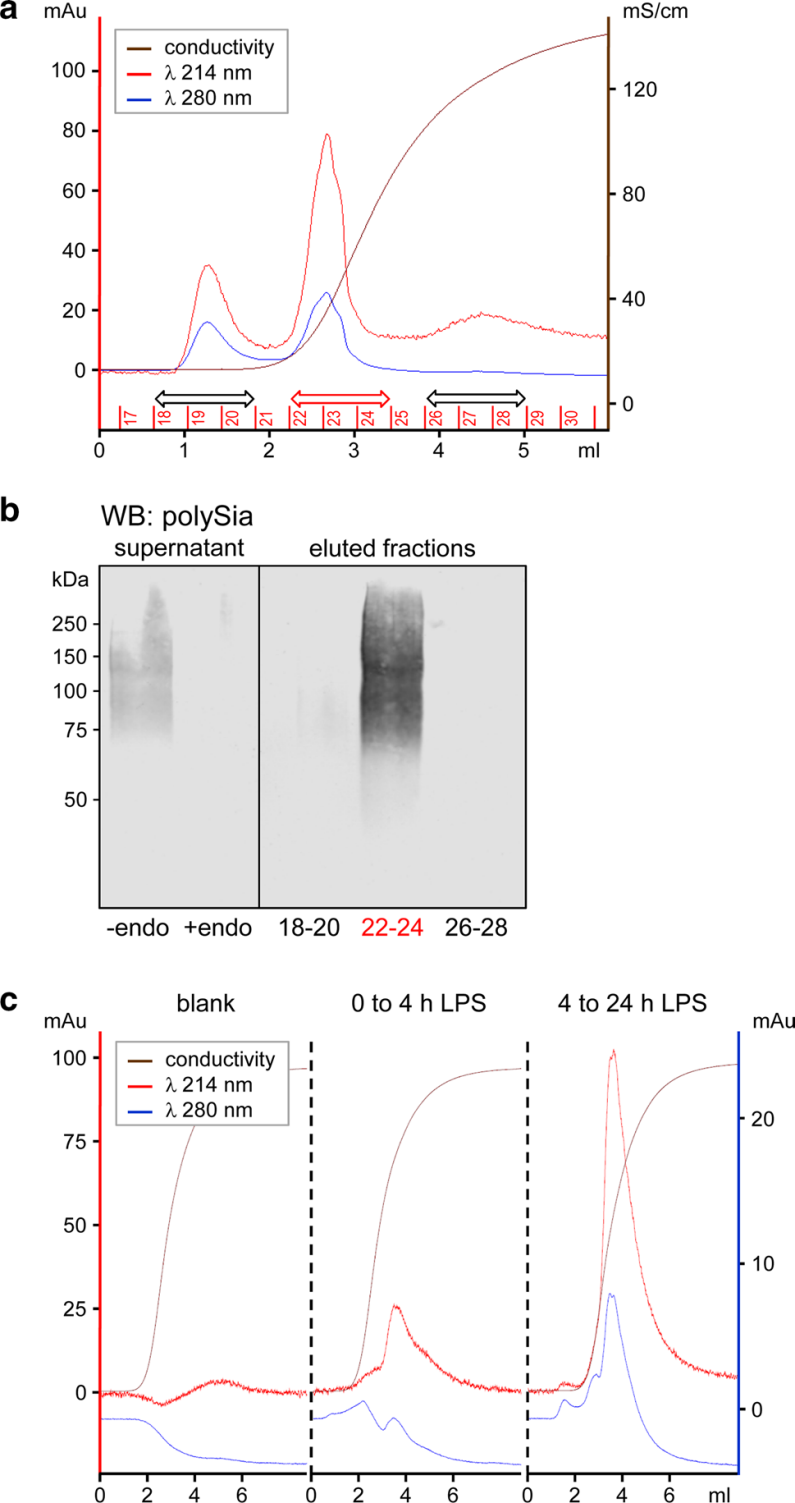
Detection of polysialylated proteins released by LPS-induced BV2 microglia. **a** Elution profile of cell culture supernatants collected from 2.5 × 10^7^ BV2 cells treated with 10-µg/ml LPS for 24 h and applied to immunoaffinity chromatography with polySia-specific antibody. The increase of conductivity (brown line) denotes the onset of elution with 2-M NaCl. Detection at 214 nm and 280 nm (red and blue line), indicative for the presence of sialic acid and protein, respectively, resulted in peaks during washing (fractions 18–20) and during elution (fractions 22–24). **b** Western blot detection of polysialylated protein in the cell culture supernatant prior to immunopurification (left panel) and in the pooled fractions 22–24, but not in fractions 18–20 and 26–28 of the chromatogram shown in **a** (right panel). Specificity of polySia detection in the supernatant was controlled by enzymatic removal of polySia with endosialidase (+ endo). **c** Elution profile of cell culture supernatants as in **a**, but collected from 3 × 10^7^ BV2 cells during the first 4 h or, after changing the medium, between 4 and 24 h after the onset of LPS treatment, respectively. The left panel shows an elution profile without sample (blank)

When analyzed by IF, polySia immunoreactivity of LPS-induced microglia completely vanished within 2 h and polysialylated proteins could be retrieved by IP from large amounts of cell culture supernatant followed by Western blot detection [5]. To monitor the release into the extracellular space, we established the sensitive detection of polysialylated proteins in cell culture supernatants of LPS-treated BV2 cells by immunoaffinity chromatography with polySia-specific antibody. Western blot analysis confirmed the presence of polysialylated protein in the pooled fractions corresponding to the absorption peak at *λ* = 214 nm obtained after the onset of the elution by a salt gradient (fractions 22–24; Fig. 3a, b). In contrast to the highly sensitive detection of polySia by immunoblotting, yields were too low to also verify the presence of ESL-1 or NRP2 in these fractions. Importantly, no polySia was detected in later fractions (26–28) or in the fractions showing similar absorption peaks during washing of the column (fractions 18–20), corroborating the specific detection of polysialylated protein. With this system, a moderate peak could be detected in supernatants of BV2 cells collected during the first 4 h after LPS induction (Fig. 3c, middle). Unexpectedly, for supernatants collected between 4 and 24 h after the onset of LPS treatment a much larger peak was obtained (Fig. 3c, right). As a rough estimate for the amount of protein-bound polySia released by LPS-induced BV2 microglia, the peaks at 214 nm, indicative for polySia, were compared to respective peaks obtained by application of defined amounts of the polySia fraction used at 500 ng/ml to inhibit the LPS-induced NO production (see Fig. 1e). As a result, approximately 400 ng of polySia could be detected in 1-ml cell culture supernatant obtained from 10^6^ LPS-induced BV2 cells after 4 h, indicating that the release of protein-bound polySia and the amount of experimentally added polySia were in a similar range. Importantly, as estimated from the peak areas, roughly 5-times more polySia-bearing protein accumulated in the cell culture supernatant during the 20 h of the second incubation as compared to the first 4-h period.

The latter indicates that, despite the rapid depletion of polySia signals detected by immunocytochemistry, polysialylated proteins are continuously released over at least 24 h after inflammatory activation of microglia. The amount of released polySia, therefore, can be much higher than anticipated based on immunostaining. Importantly, this also means that it will not be possible to directly detect by immunohistochemistry, if polySia is released from activated microglia into the brain parenchyma.

### Detection of polySia-positive microglia in TBI

**Fig. 4:**
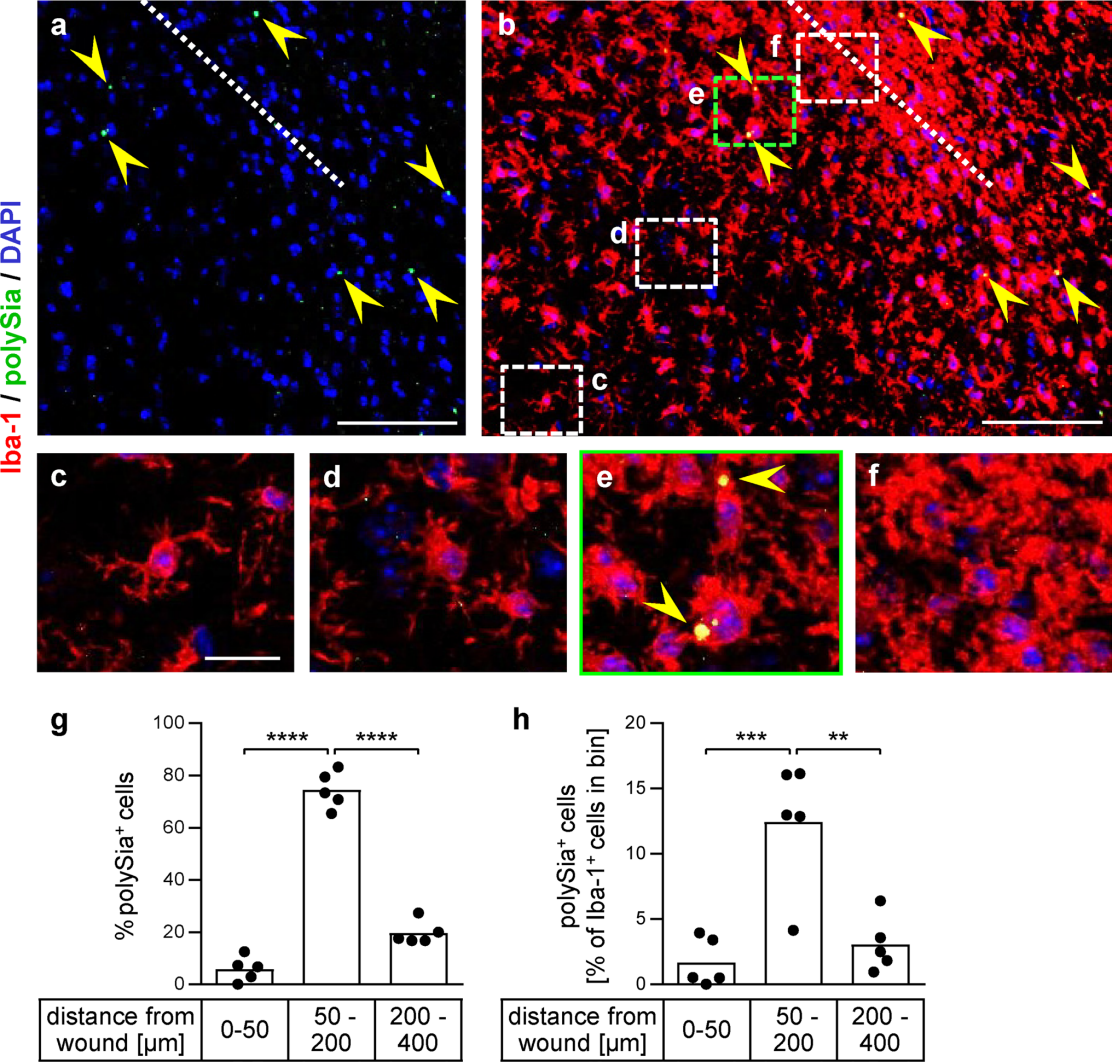
PolySia-positive microglia in TBI. Immunofluorescence detection of polySia (green, marked by yellow arrowheads) and Iba-1 (red) one week after injury by an injection through the mouse cortex. **a, b** Overview of polySia signals around the wound channel (**a**), merged with Iba-1 staining (**b**). The wound channel is indicated by a dotted line. Nuclei were counterstained with DAPI (blue). **c**–**f** Higher magnification views of the boxed areas highlighted in **b**. See text for a detailed description. Scale bars, 100 µm in **a** and **b**, 20 µm in **c**. **g, h** Quantitative assessment. Iba-1-positive cells with polySia-positive dots of at least 5 µm^2^ were counted in three bins at distances between 0 and 50, 50 and 200, 200 and 400 µm from the wound channel. A total of 134 polySia-positive cells were detected on sections of *n* = 5 lesioned brains [one section per brain evaluated, mean per section = 27 ± 8.4 (s.e.m)]. Distributions are shown in % of polySia-positive cells (**g**) and in % of all Iba-1-positive cells detected in the evaluated areas (**h**). Per bin, the individual values and means are plotted. Repeated measure one-way ANOVA indicated significant differences and results from Tukey’s post hoc test are shown (**p < 0.01, ***p < 0.001, ****p < 0.0001)

The presence of polySia in activated microglia after TBI was addressed in adult mice seven days after an injection of kainate through the cortex into the CA1 sector of the dorsal hippocampus. Serial sections of paraffin-embedded brain from five lesioned animals were investigated by staining for polySia and Iba-1. As shown before, the hippocampal kainate injection causes microglia activation in the hippocampus and adjacent cortical areas but, apart from the wound channel, not in the outer cortical layers [24]. Therefore, microglia around the wound channel was evaluated only in superficial areas of the cortex. All five specimen displayed the polySia and Iba-1 patterns of the representative section shown in Fig. 4. In areas distant from the wound channel (indicated by the dotted line in Fig. 4a, b), Iba-1 staining revealed microglia with ramified morphology indicative for the quiescent state [36, 37] (Fig. 4b and detail in c). Closer to the lesion, microglia had shorter and thicker processes (Fig. 4d), characteristic for the transition into a reactive state in response to injury; whereas, rounded, amoeboid cells without defined processes at the site of the lesion indicate a fully activated state of phagocytic microglia (Fig. 4f). Notably, only a few polySia-positive dots were detected in some distance around the wound located in microglia with rounded morphology (Fig. 4a, b, e; for a quantitative assessment, see Fig. 4g, h). Enzymatic removal of polySia with endosialidase eliminated all of these dots in Iba-1-positive cell, but not some of the scattered smaller signals, which, therefore, are considered to be nonspecific (see Online Resource 2). Attempts to co-stain with the Siglec-E-specific antibody successfully used for immunocytochemistry (see below) or with the Golgi marker giantin failed, but the dot-shaped cellular pattern corresponds to the appearance of Golgi-localized polySia during the lesion-induced activation of microglia in acute brain slice cultures [5]. The distribution in a halo around the wound channel indicates accumulation of polySia in the Golgi of injury-induced microglia, which is lost during further activation. Analogous to the continuous release of polySia after loss of Golgi-localized polySia by inflammatory activation of microglia in vitro, this pattern suggests polySia shedding by injury-activated microglia in the brain parenchyma during TBI in vivo.

### Diverging mRNA and protein patterns of Siglec-E upon LPS induction

**Fig. 5:**
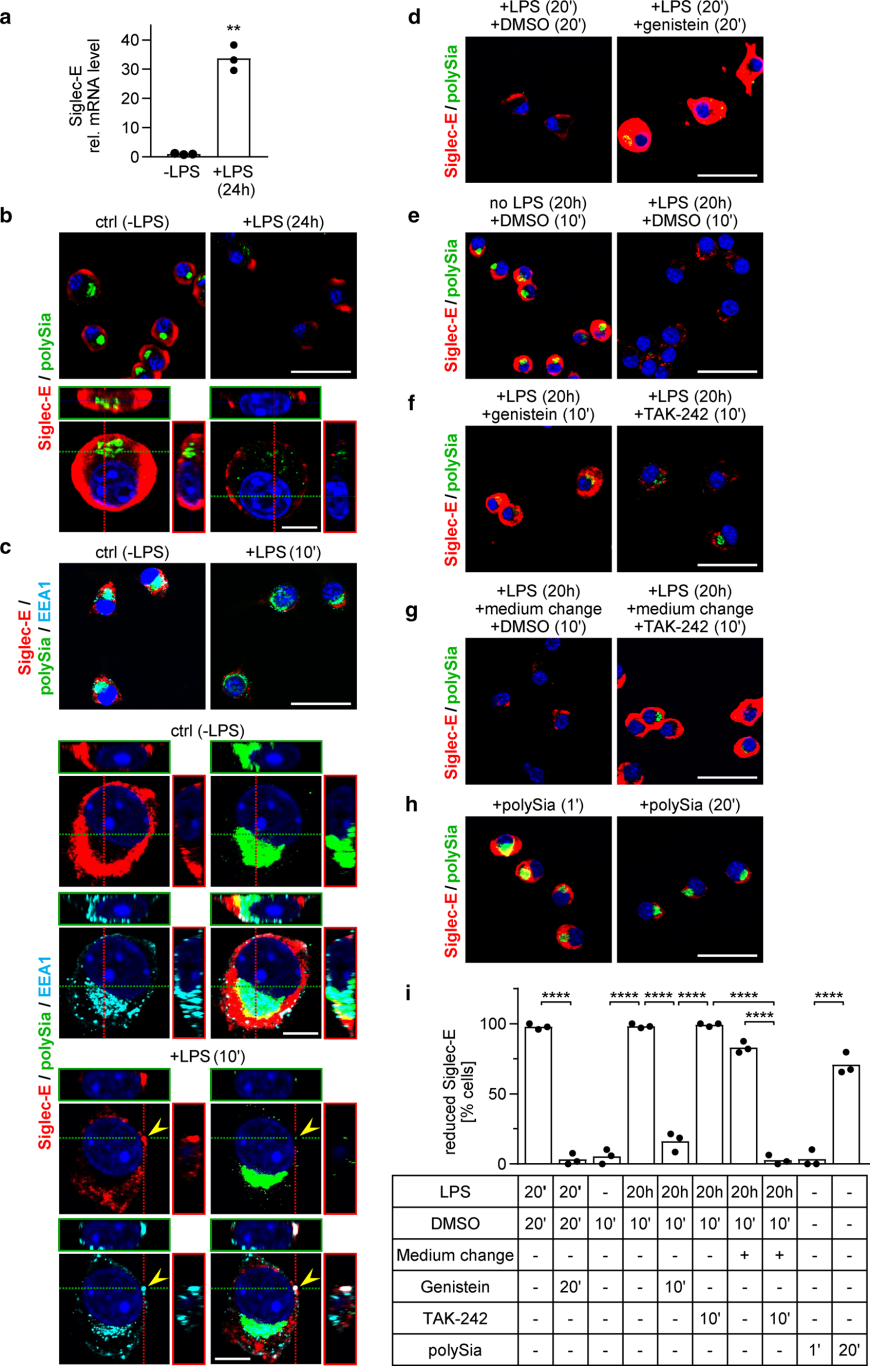
LPS-induced changes of Siglec-E in BV2 microglia. **a** Analysis by quantitative real-time RT-PCR reveals a strong increase of Siglec-E mRNA in cells treated with 1-µg/ml LPS for 24 h. Individual values and means from 3 independent treatments per group are plotted. **p < 0.01, unpaired *t* test. **b** Immunofluorescence detection of Siglec-E (red) and polySia (green). Nuclear counterstain with DAPI (blue). Staining patterns before (control, ctrl) and after treatment with 1-µg/ml LPS for 24 h. Lower panels show 3D reconstructions at higher magnification. **c** Colocalization of Siglec-E (red) and polySia (green) with the endosomal marker EEA1 (cyan). Staining patterns before (control, ctrl) and after treatment with 1-µg/ml LPS for 10 min. Lower panels show 3D reconstructions of single and merged channels with nuclear counterstain (DAPI, blue) at higher magnification. **d**–**h** Detection of Siglec-E (red) and polySia (green) as in **b**. **d** Staining patterns after 20 min of LPS treatment in the presence of solvent (1-µl/ml DMSO, left) or 200-µM genistein (right). **e**, **f** Staining patterns after incubation for 20 h without or with LPS followed by 10 min with 1-µl/ml DMSO (**e**), 200-µM genistein, or 1-µM TAK-242 (**f**), as indicated. Genistein and TAK-242 were added as 1-µl/ml stock solution in DMSO. **g** Incubation of LPS-treated cells with DMSO and TAK-242 as in **e** and **f**, but this time the cell culture medium was changed to apply DMSO and TAK-242. **h** Staining patterns after incubation with polySia (10 µg/ml) for 1 min (left) or 20 min (right). The strong Siglec-E signals under control conditions without LPS (**b**, **c**, **e**, **h**) and after genistein treatment (**d**, **f**) are overexposed to enable a visualization of the weak signals in LPS-treated cells with the same camera setting. Scale bars, 50 µm in **b** and **c** (upper panels) and in **d**–**h**; 10 µm in **b** and **c,** lower panels. **i** Quantitative assessment of reduced Siglec-E cell surface staining under the conditions shown in **d**–**h**. Based on the densitometric evaluation of signal intensities of 108 cells treated for 10 min with DMSO in the absence of LPS, intensities below 50% of the mean Siglec-E signal intensity under these conditions were considered “reduced”. Per well, a minimum of 20 cells in 3 randomly selected frames with at least 5 cells each were evaluated and the percentage of cells with reduced Siglec-E immunoreactivity was calculated. Individual values and means of 3 wells per condition are plotted. One-way ANOVA indicated significant differences (*p* < 0.0001) and results from Tukey’s post hoc test are shown for selected comparisons (****p < 0.0001)

In accordance with the previous reports on bone marrow-derived macrophages [38, 39], Siglec-E mRNA expression levels were strongly increased in LPS-treated BV2 microglia (Fig. 5a). The evaluation of Siglec-E protein patterns by IF, however, revealed abundant Siglec-E immunoreactivity on the surface of untreated cells (Fig. 5b, left), which was drastically reduced and restricted to a few immunoreactive patches after 24 h of LPS treatment (Fig. 5b, right). 3D reconstruction at higher magnification (Fig. 5b, lower panels) not only corroborated the strong reduction of Siglec-E at the cell surface and the loss of perinuclear polySia, but also revealed some small polySia-immunoreactive puncta within the LPS-treated cells, which may represent export vesicles engaged in continuous shedding of polysialylated protein.

Based on the discrepancy between Siglec-E mRNA and protein detection, and because Siglecs can be internalized upon ligand binding or antibody-mediated crosslinking [38, 40], we investigated if LPS induction leads to endocytosis of Siglec-E. After 10 min of LPS treatment, Siglec-E immunoreactive dots were detected within the cells and some, but few of them colocalized with EEA1, a well-characterized marker of early endosomes in clathrin-dependent and -independent pathways of endocytosis [41] (Fig. 5c). In some cases, polySia colocalized with the EEA1- and Siglec-E-positive vesicle-like structures but most of the abundant polySia-positive puncta are not colocalized with these markers, most likely because these structures denote secretory vesicles delivering the polysialylated proteins from the Golgi to the cell surface. In contrast to the strong reduction of Siglec-E after LPS treatment, incubation for 20 min with LPS in the presence of genistein, an inhibitor of clathrin-independent endocytosis [42], caused a massive accumulation of Siglec-E at the cell surface (Fig. 5d; for a quantitative assessment, see Fig. 5i). A comparable accumulation was achieved by genistein treatment of cells that were previously activated with LPS for 20 h (Fig. 5e, f, i). Notably, genistein may also interfere with LPS-induced TLR4 activation [43], but selective inhibition of TLR4 signaling by application of TAK-242 [44] to the cell culture supernatant of LPS-induced cells for 10 min was not causing cell surface accumulation of Siglec-E (Fig. 5f, right; i). However, similar to genistein, TAK-242 treatment led to reappearance of intracellular, perinuclear polySia in some of the cells. Assuming that the internalization of Siglec-E is caused at least in part by its interactions with the protein-bound polySia released by LPS-induced BV2 microglia and that the reappearance of perinuclear polySia signals indicates a reduction of this release, the internalization of Siglec-E should come to a halt, if TAK-242 was applied after removal of the cell culture supernatant containing previously released polySia. Indeed, recurrence of Siglec-E signals at the surface of LPS-treated cells was observed, when TAK-242 was added by replacing the cell culture medium (Fig. 5g, i). To directly test if internalization of Siglec-E can be induced by interactions with polySia, protein-free polySia was applied to BV2 microglia. After 20 min of incubation, Siglec-E signals at the surface of otherwise untreated cells were clearly reduced (Fig. 5h, i). Short-term exposure for 1 min had no such effect, ruling out the possibility that engagement of Siglec-E with polySia blocks its recognition by the Siglec-E-specific antibody used in the current study.

Together, these data reveal that Siglec-E is continuously produced in LPS-induced BV2 microglia, which is consistent with the high Siglec-E mRNA levels of LPS-treated cells. The data also indicate that polySia release and Siglec-E internalization are regulated by different, TLR4-dependent and TLR4-independent pathways, respectively, and provide first evidence that Siglec-E is internalized in response to polySia as a *trans*-interacting ligand.

### Knockout of Siglec-E potentiates LPS-induced activation and eliminates the response to polySia

**Fig. 6:**
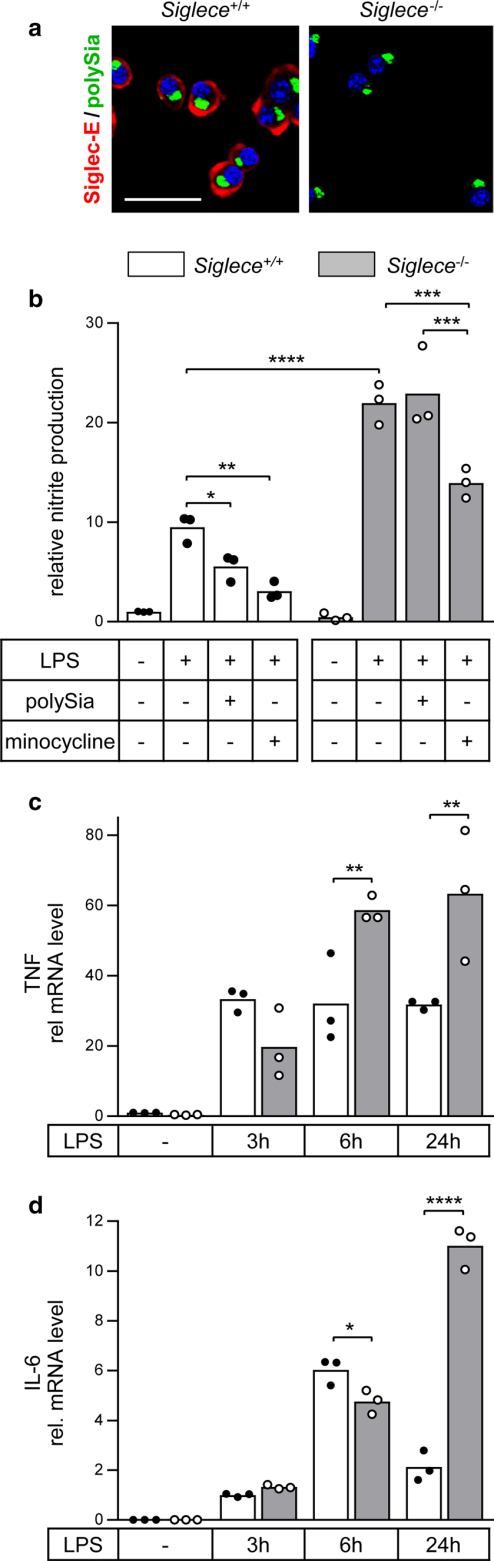
Loss of Siglec-E abrogates responsiveness to polySia and enhances LPS-induced activation. **a** Compared to wildtype BV2 cells (*Siglece*^+/+^), the immunoreactivity of Siglec-E (red), but not polySia (green) is abolished by CRISPR/spCas9-mediated knockout of *Siglece* (*Siglece*^−/−^, clone D19). Nuclear counterstain with DAPI (blue). Scale bar, 50 µm. **b** Comparable to the effect of preincubation with 60-µM minocycline for 2 h, application of polySia (5 µg/ml) inhibits the LPS-induced NO production of *Siglece*^+/+^ but not *Siglece*^−/−^ BV2 microglia (clone D19). In addition, the LPS-induced NO production of *Siglece*^−/−^ microglia was significantly higher. Where indicated (LPS +), 1-µg/ml LPS was applied for 24 h. **c**, **d** During 24 h of LPS treatment, Siglec-E-negative cells also showed a significantly more pronounced increase of TNF and IL-6 mRNA levels. In **b**–**d**, individual values and means from *n* = 3 independent treatments per group are plotted. Mixed two-way ANOVA indicated significant differences and results from Holms–Sidak post hoc test are shown for selected group comparisons (*p < 0.05, **p < 0.01, ***p < 0.001, ****p < 0.0001)

CRISPR/spCas9-mediated genome editing was used to generate a single Siglec-E-negative BV2 cell clone (clone D19, Fig. 6a, see Online Resource 3 for sequence analysis of induced mutations) and a mixed pool of four other Siglec-E-negative clones (see Online Resource 4 for IF analysis of Siglec-E loss). These cells were used to corroborate if Siglec-E is accountable for the response of microglia to externally applied polySia and to address a role of Siglec-E in the proposed negative feedback inhibition of LPS-induced microglia activation by the shedding of polysialylated proteins. Consistent with our previous study, showing potentiated LPS induction of polySia-negative microglia [4], proinflammatory activation was initially monitored by the detection of NO-derived nitrogen species with the Griess assay.

Comparative analysis of wildtype (*Siglece*^+/+^) and Siglec-E-deficient BV2 cells (*Siglece*^−/−^, clone D19) revealed that both responded to LPS with a strong increase in NO production (Fig. 6b). This response could be inhibited in both lines by preincubation with minocycline, a widely used inhibitor of proinflammatory microglia polarization [45]. In contrast, the attenuation of the LPS-induced increase by addition of exogenous polySia was abolished in the Siglec-E-deficient line and the response towards LPS was substantially stronger. To test for the possibility of an unspecific clonal effect as the basis for the altered responsiveness, the experiment was repeated with a mixture of another four *Siglece*^−/−^ clones. As shown in Online Resource 5, the outcome was basically the same, i.e., a dramatically higher LPS-induced NO production than in wildtype cells that could still be inhibited by preincubation with minocycline, but no longer by the addition of polySia. Furthermore, the Siglec-E-negative clone D19 displayed a significantly more pronounced increase in the mRNA levels of the proinflammatory cytokines tumor necrosis factor (TNF) and interleukin-6 (IL-6) over 24 h of LPS treatment, although there was a slight delay in upregulation during the early phase of LPS induction (Fig. 6c, d). Taken together, these findings indicate that the release of microglia-intrinsic polySia attenuates the inflammatory response by acting as a *trans*-activating ligand of Siglec-E.

## Discussion

The current study provides the first indication that injury-induced activation leads to intracellular accumulation of poly—Sia in murine microglia in vivo. Comparable to the delayed and slowly increasing appearance of Golgi-localized polySia during microglia activation in acute brain slices over five days of culture [5], Iba-1-positive cells with strong perinuclear polySia signals featuring the morphology of activated microglia were detected seven days after brain lesion. These cells were rare and located at least 50-µm deep in the parenchyma around the wound channel, indicating that they are microglia rather than infiltrating macrophages. Although microglia activation peaks between three and five days after TBI [46], it has been shown in a rat TBI model that microglia acquire a predominantly proinflammatory activation phenotype at seven days post-injury [47]. We, therefore, assume that cultured microglia, which consistently display an accumulation of polySia in the Golgi compartment, correspond to a transient activation state of polySia-positive microglia in TBI. In analogy to the continuous shedding of polysialylated proteins by activated microglia in vitro, the absence of polySia signals in fully activated microglia situated directly adjacent to the lesion points towards a release of polySia. Furthermore, the absence of Golgi-confined polySia in ramified microglia located more distant from the lesion site confirms respective findings on quiescent microglia in murine brain slice cultures [5]. As a proof of principle, these data indicate that polySia can be produced by activated microglia in vivo. Future studies should follow up the time course of its appearance and ask for its potential impact on pathological microglia activation as well as on the outcome of TBI.

Our in vitro experiments with murine BV2 microglia corroborate data from primary and stem cell-derived microglia by demonstrating that polySia is presented on the two carrier proteins ESL-1 and NRP2, which, based on a glycoproteomic analysis of stem cell-derived microglia, constitute the pool of Golgi-confined polysialylated proteins in these cells [5], and that LPS-induced inflammatory activation causes a rapid translocation of polySia to the cell surface [5]. Consistent with the LPS-induced mobilization of internal calcium stores through activation of RyRs [34, 48], the release from the Golgi could be mimicked by treatment with the RyR agonist 4-C*m*C; whereas, inhibition by the corresponding antagonist DHBP prevented the LPS-induced translocation. Notably, the trans-Golgi compartment, where polysialylation takes place, behaves as a calcium store that can be selectively mobilized by RyRs [49, 50]. Thus, calcium depletion initiates the release of polySia-ESL-1 and polySia-NRP2 by abrogating their retention in the Golgi compartment.

As shown before, polySia-negative ESL-1 maintains its Golgi localization in LPS-induced microglia; while, polySia-negative NRP2 is distributed over the entire cell before and after activation [4, 5]. Therefore, and in the absence of any other obvious similarity between NRP2 and ESL-1, polysialylation is likely to form the basis for the common regulation of Golgi retention and release. ST8SIA4, responsible for polysialylation of ESL-1 and NRP2 in microglia [4, 5], interacts with the nascent polySia chains and with the protein acceptors [51, 52]. Since calcium affects interactions of polySia with proteins, probably by stabilizing conformational epitopes of polySia [53], interactions of polySia with ST8SIA4 could mediate the calcium-dependent retention of polySia-NRP2 and poly—Sia-ESL-1 in the Golgi. Alterations of these interactions caused by decreasing calcium levels could, therefore, trigger their discharge. Alternatively, or in addition, other poly—Sia-binding factors could engage in calcium-dependent mechanisms that regulate cargo sorting and exit from the trans-Golgi compartment [54].

In contrast to the complete loss of cell-associated polySia after 20 min of LPS induction, which is due to metalloproteinase-dependent ectodomain shedding of polySia-ESL-1 and polySia-NRP2 [5], the accumulation of polySia at the cell surface of 4-C*m*C-treated cells persisted for at least 20 min. This indicates that the mere presentation of the polysialylated proteins at the cell surface as caused by the RyR-dependent manipulation of internal calcium levels is not sufficient for ectodomain shedding. Instead, the responsible sheddase must be recruited and/or activated by an alternative mechanism. Although it is well known that a number of key metalloproteinases are upregulated during LPS-induced microglia activation [55, 56], the rapid depletion of cell-associated polySia precludes a transcriptional regulation.

Only recently, first evidence became available that Siglec-E is able to bind polySia and that knockdown of Siglec-E abolishes the response of cultured microglia to experimentally applied polySia [9, 17]. In contrast to a glycan array study indicating a manifold stronger binding of Siglec-E to α2,8-linked di- and trisialic acid as compared to oligosialic acids with DPs between 4 and 11 [16], we now demonstrate that application of DP3 to murine microglia cannot mimic the physiological effect of polySia (colominic acid) with an average DP of about 50. Similarly, only polySia with an average DP of 20, but not oligosialic acid with DP6 was able to inhibit LPS-induced activation of human THP-1 macrophages [8], although studies with Siglec-11-Fc chimera indicated binding of short oligomers [15]. The reasons for these discrepancies between binding and physiological effects remain to be further explored. However, in analogy to the observation that multivalent display of DP2 on nanoparticles, but not soluble DP2, causes Siglec-E clustering and inhibits LPS-induced macrophage activation [57], it appears likely that only polySia but not soluble DP3 is able to induce Siglec-E oligomerization and thereby inhibitory signaling.

By the use of CRISPR/spCas9-mediated knockout, we corroborate that experimentally applied polySia is no longer able to inhibit LPS-induced activation of Siglec-E-deficient microglia. Importantly, we also show that Siglec-E-negative microglia exhibit a marked increase of activation in response to LPS. This is highly reminiscent of the augmented LPS induction of microglia obtained from ST8SIA4-deficient mice, i.e., microglia that are not able to produce and release polySia [4]. Thus, in the absence of any external source of polySia, the deletion of the putative polySia receptor has the same effect as the elimination of polySia biosynthesis, i.e., the cell-intrinsic source of the assumed ligand. Taken together, these findings imply that shedding of polysialylated proteins is part of a mechanism for negative feedback inhibition of LPS-induced microglia, in which the released protein-attached polySia activates Siglec-E as a ligand interacting in *trans*. Consistent with this model, LPS-induced ST8SIA4-negative microglia are still responsive to experimentally applied polySia indicating uncompromised sensing [4]. Conversely, the LPS-induced activation of Siglec-E-deficient microglia can still be inhibited by minocycline, proving that the responsiveness to unrelated anti-inflammatory treatment is maintained (this study).

Regarding the spatiotemporal distribution of Siglec-E immunoreactivity, the strong reduction of Siglec-E on LPS-induced microglia stands in stark contrast with the strong up-regulation of Siglec-E on the mRNA level. The strong reduction of Siglec-E also appears contradictory to the finding that Siglec-E is required for polySia-mediated inhibition. However, these data might be explained by endocytosis of Siglec-E upon ligand binding, which seems to be a general feature of Siglecs [11]. Indeed, intracellular Siglec-E staining and colocalization with the early endosomal marker EEA1 as well as with polySia could be detected during the early phase of LPS induction. Furthermore, inhibition with genistein for 10 or 20 min not only prevented the reduction, but also caused a progressive accumulation of Siglec-E at the surface of LPS-treated BV2 microglia, even when applied after 20 h of LPS induction. This is comparable to the inhibitory effect of genistein on the clathrin-independent endocytosis of Siglec-F, another CD33-related murine Siglec, trafficking to lysosomes for degradation, when induced by anti-Siglec-F antibody ligation [40]. Selective inhibition of TLR4 signaling by TAK-242 reinstated the intracellular accumulation of polySia but in contrast to the effect of genistein, TAK-242 was only able to restore Siglec-E on the surface of LPS-induced cells when applied together with a change of the cell culture medium. This indicates that the internalization of Siglec-E in LPS-induced microglia is regulated differently from the release of polysialylated protein and not caused by TLR4 signaling or *cis*-interactions with TLR4, as implicated by previous work on the role of Siglec-E in the endocytosis of activated TLR4 [58, 59]. Our data rather support the assumption that Siglec-E is internalized by receptor clustering and/or activation induced by interactions with shed polySia in *trans.* This is compatible with a recent report concluding that the inhibitory effects of Siglec-E on LPS-induced macrophages depend on activation by *trans*-interacting ligands [39].

An unexpected finding with high relevance for all future studies on possible physiological consequences of polysialylated protein shedding was that the release is not just a one-time discharge of polySia carriers assembled in the Golgi compartment of cultured microglia. Instead, production and release appear to continue for at least 24 h after LPS induction in vitro and a rough estimate of the released amounts indicated that they are sufficient to exert inhibitory effects. Correspondingly, the presence of polySia in a small proportion of microglia in some distance around a brain lesion and its absence from highly activated microglia in direct vicinity to the lesion site suggest that polysialylated proteins, after a transient accumulation in the Golgi, are continuously released by these cells in vivo, although it is not possible to directly detect this by immunohistochemistry. A major goal of future experiments, therefore, will be to develop appropriate sampling and analytical methods to detect soluble polySia-NRP2 and polySia-ESL-1 in freshly isolated brain tissue following TBI.

The termination of inflammatory activation by inhibitory, ITIM-containing receptors is an essential component of a proportionate and effective immune response [60]. In this regard, counteracting the inflammatory activation of microglia by shedding of polySia-presenting proteins may be beneficial for the outcome of TBI. Furthermore, externally applied polySia might be a therapeutic avenue to potentiate the anti-inflammatory effect in TBI, similar to current approaches to inhibit the activation of microglia and infiltrating macrophages, for instance, by application of minocycline, or by therapeutic modulation of TLR signaling [46, 61, 62]. Minocycline, however, not only reduces chronic microglial activation after brain trauma but also leads to increased neurodegeneration [63], indicating the need for a balanced approach to modulate neuroinflammation [64]. Notably, externally added polySia is as effective as minocycline in inhibiting inflammatory activation in primary and stem cell-derived [4, 5] as well as in BV2 microglia (this study). Future studies should, therefore, explore the role of polySia shedding in TBI as well as the effects of externally added polySia on injury-induced microglia.

## Electronic supplementary material

Below is the link to the electronic supplementary material.

Supplementary file1 (PDF 2275 kb)
