# Supplementary material for: Polysialic acid and Siglec-E orchestrate negative feedback regulation of microglia activation
Source: Cell Mol Life Sci. 2020 Jul 28;78(4):1637–53. doi: 10.1007/s00018-020-03601-z (PMC7904730; doi:10.1007/s00018-020-03601-z)
Supplement: Supplementary file 1 — Supplementary file1 (PDF 2275 kb) [file 18_2020_3601_MOESM1_ESM.pdf]

*Cellular and Molecular Life Sciences*

# Polysialic acid and Siglec-E orchestrate negative feedback regulation of microglia activation

Hauke Thiesler, Julia Beimdiek, and Herbert Hildebrandt

**Corresponding author**:

Herbert Hildebrandt, Institute of Clinical Biochemistry, Hannover Medical School,

E-mail: hildebrandt.herbert@mh-hannover.de

**Supplementary material:**

Online Resource 1: Analysis of the polySia chain lengths pattern

Online Resource 2: Endosialidase treatment eliminates polySia signals in injury-induced microglia

Online Resource 3: Analysis of CRISPR/spCas-9 induced mutations of *Siglece* in BV2 cell clone D19

Online Resource 4: Loss of Siglec-E in a mixed population of four different *Siglece*$^{-/-}$ BV2 cell clones

Online Resource 5: Altered LPS-induced NO production and loss of polySia responsiveness in a mixed population of four different *Siglece*$^{-/-}$ BV2 cell clones

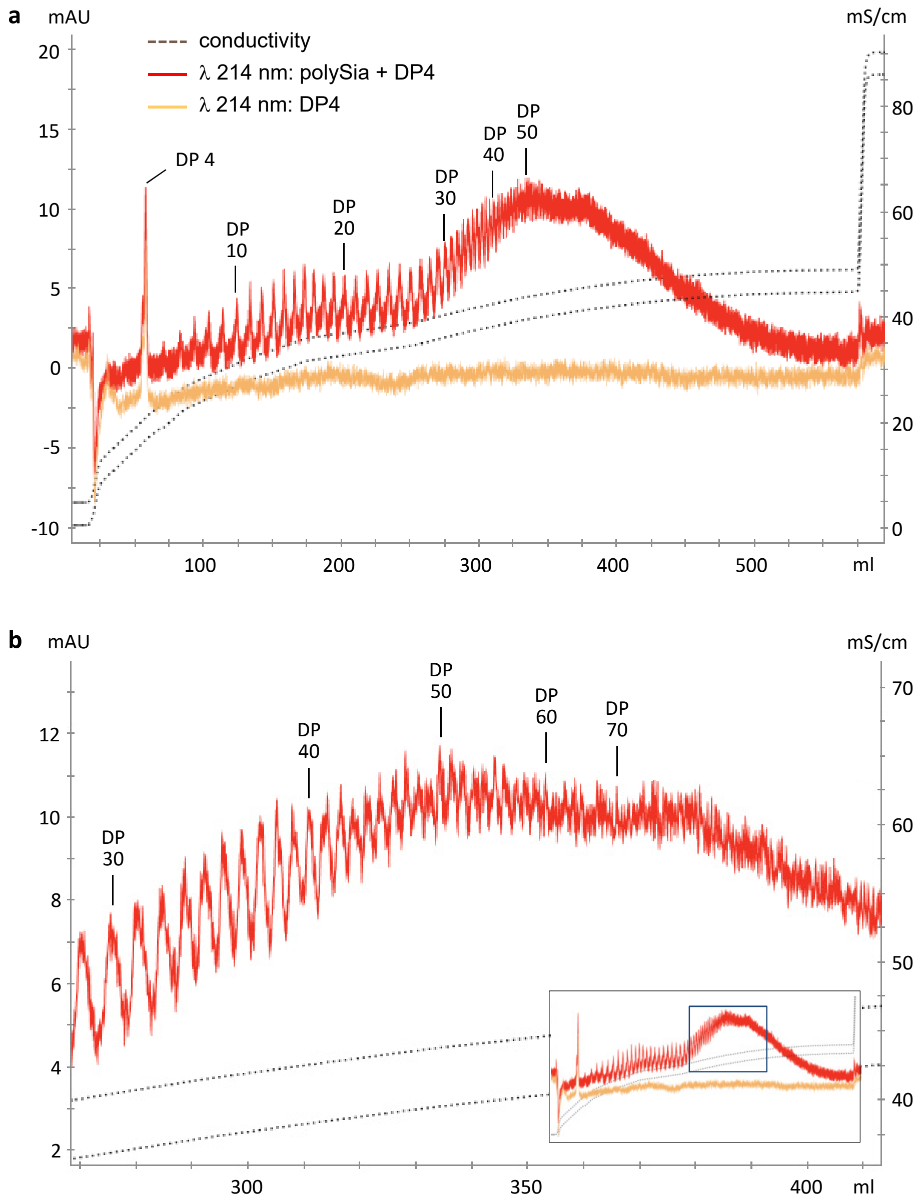


## Online Resource 1

Anion exchange chromatography of the polySia batch used in the current study (colominic acid, Sigma-Aldrich, lot no. 110M1383). The degree of polymerization (DP) of the sialic acid polymers was determined by spiking with α2,8-linked tetrasialic acid (DP4) as a standard. The *N*-acetyl groups of sialic acid were detected by absorption at 214 nm.

**a** The elution profile of polySia spiked with DP4 (red) is shown in comparison with a blank run spiked with DP4 alone (orange). Elution by NaCl gradients was monitored by conductivity (dotted brown lines). Peaks of selected DPs are labeled.

**b** Enlarged view of the profile segment highlighted by the boxed area of the insert.

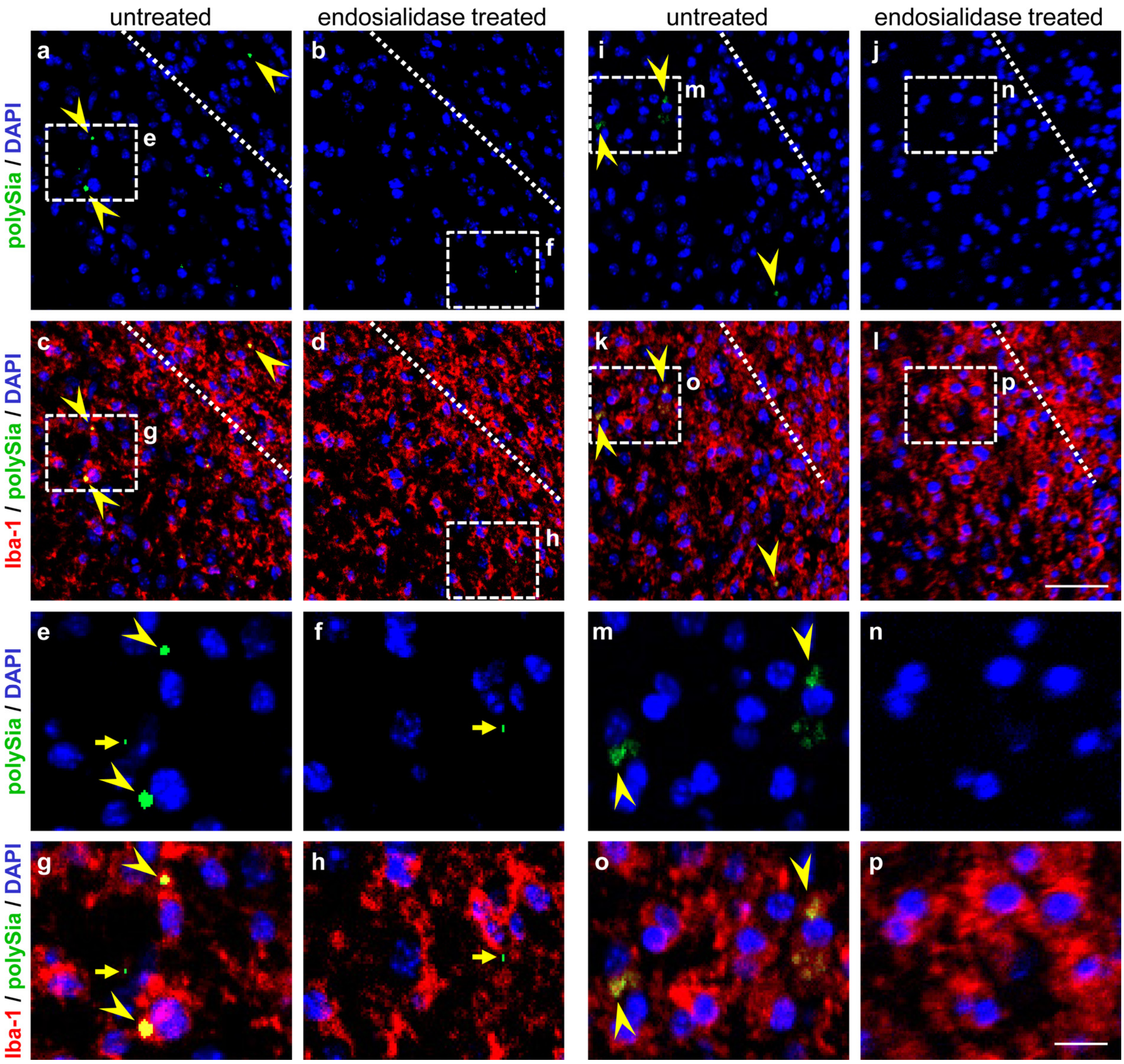


**Online Resource 2** Endosialidase treatment eliminates polySia signals in injury-induced microglia.

Immunofluorescence detection of polySia (green) and Iba-1 (red) in the cortex of two different mice one week after injury by an injection through the cortex. Images of untreated sections and of consecutive sections from the same specimen treated with the polysialic acid-specific enzyme endosialidase prior to immunostaining, as indicated. Images in the left column (**a, c, e ,g**) are from the section shown in Fig. 4. Upper part: Overviews with polySia signals around the wound channel (**a, b, i, j**), merged with Iba-1 staining (**c, d, k, l**). Nuclei were counterstained with DAPI (blue). The wound channel is indicated by a dotted line. Lower part: Higher magnification views of the boxed areas. Iba-1 positive cells with polySia-positive dots > 5 µm$^2$ are indicated by arrowheads. Arrows in **e** and **f** indicate examples of small signals that were not eliminated by endosialidase treatment and therefore considered unspecific. See text to Fig. 4 for further description. Scale bars, 50 µm in **l**, 20 µm in **p.**

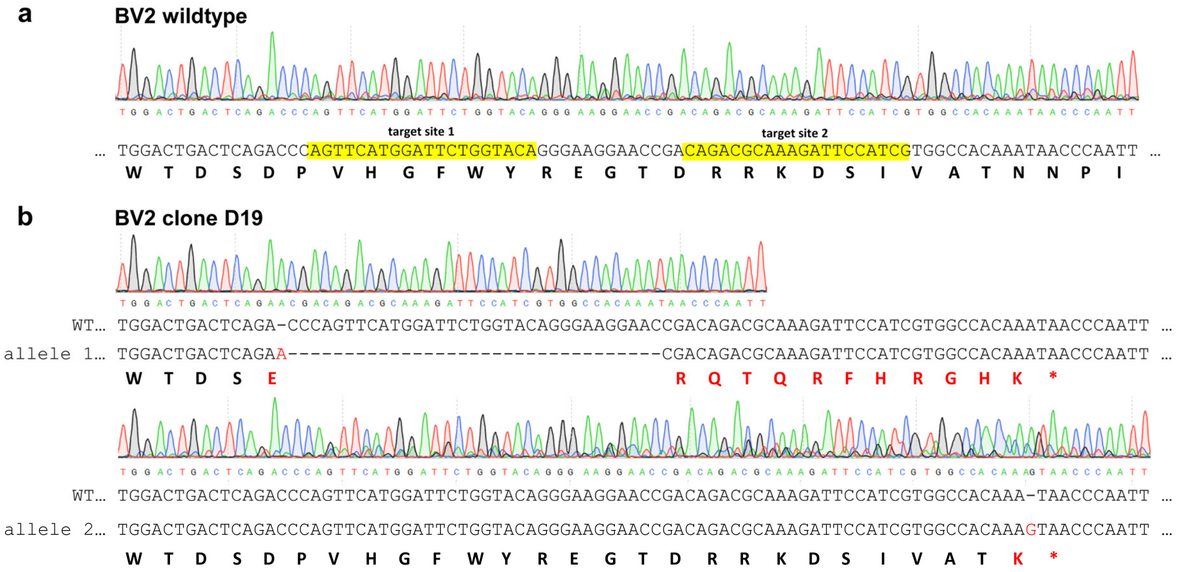


**Online Resource 3** Sequencing analysis of the sialic acid binding V-domain within the first exon of *Siglece* in BV2 cell clone D19 generated by CRISPR/spCas9-mediated genome editing.

**a** Sequencing of wildtype BV2 cells. Guide RNA binding sites are highlighted.

**b** Frameshifts and premature stop codons (*) due to nucleotide insertions (red) in both alleles and a 33 bp deletion in allele 1 of clone D19.

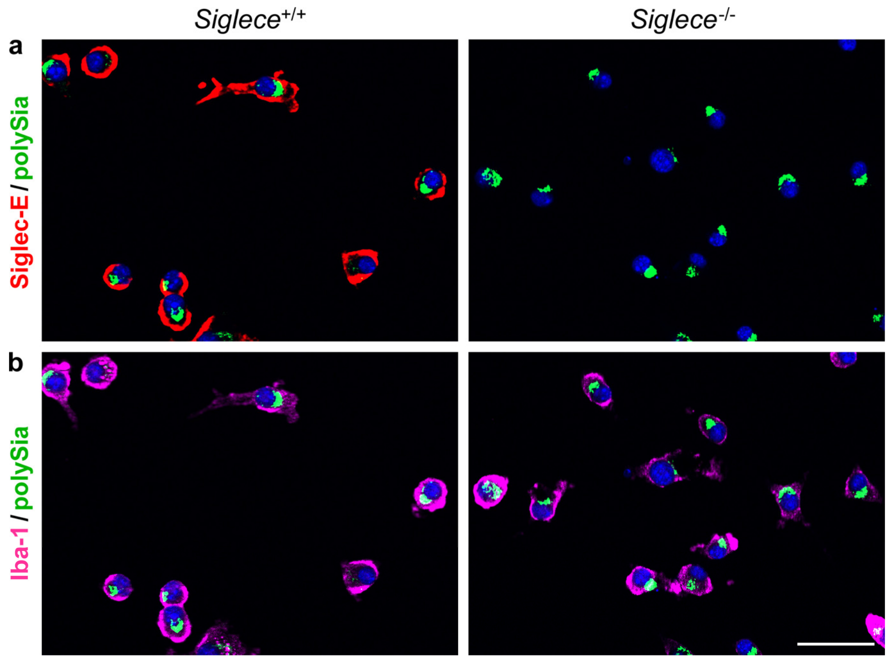


**Online Resource 4** Loss of Siglec-E in the mixed population of four different *Siglece*$^{-/-}$ BV2 cell clones.

**a** Immunoreactivity of Siglec-E (red) and polySia (green) in parental BV2 cells (*Siglece*$^{+/+}$) and the mixed *Siglece*$^{-/-}$ clones.

**b** Immunoreactivity of Iba-1 (magenta) and polySia (green) of the cells shown in **a**.

Nuclear counterstain with DAPI (blue). Scale bar, 50 µm.

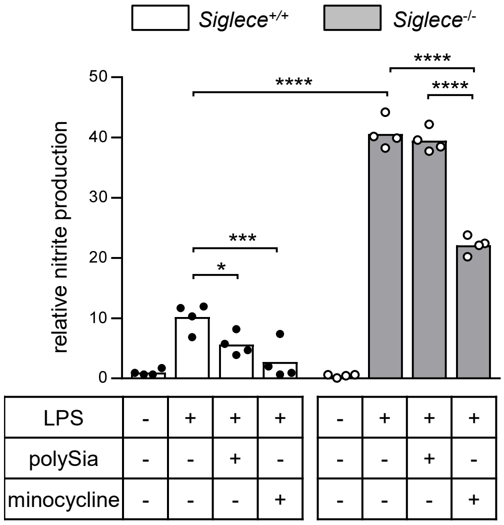


**Online Resource 5**

A mixed population of four different *Siglece*$^{-/-}$ BV2 cell clones reproduces the changes of LPS-induced NO production as shown in Fig. 6 for a single *Siglece*$^{-/-}$ clone.

Left: Corroborating the results shown in Fig. 6b, the NO production of Siglec-E-positive (*Siglece*$^{+/+}$) BV2 cells in response to LPS (1µg/ml applied for 24 h) was inhibited by the addition of polySia (5 µg/ml) or by preincubation with 60 µM minocycline for 2 h.

Right: Compared to the parental *Siglece*$^{+/+}$ BV2 cells, the mixture of Siglec-E-negative BV2 cell clones (*Siglece*$^{-/-}$), not including the clone used in the experiment depicted in Fig. 6b, shows a significantly higher response to LPS-induced NO production that was inhibited by preincubation with minocycline, but not by the addition of polySia.

Individual values and means from n = 4 independent treatments per group are plotted. Mixed two-way ANOVA indicated significant differences and results from Holms-Sidak post hoc test are shown for selected group comparisons (* $p$<0.05, *** $p$<0.001, **** $p$<0.0001).
